# Systematic identification and quantification of phase variation in commensal and pathogenic *Escherichia coli*

**DOI:** 10.1186/s13073-014-0112-4

**Published:** 2014-11-28

**Authors:** Amir Goldberg, Ofer Fridman, Irine Ronin, Nathalie Q Balaban

**Affiliations:** Racah Institute of Physics and the Sudarsky Center for Computational Biology, The Hebrew University, Edmond J. Safra Campus, Jerusalem, 91904 Israel

## Abstract

Bacteria have been shown to generate constant genetic variation in a process termed phase variation. We present a tool based on whole genome sequencing that allows detection and quantification of coexisting genotypes mediated by genomic inversions in bacterial cultures. We tested our method on widely used strains of *Escherichia coli*, and detected stable and reproducible phase variation in several invertible loci. These are shown here to be responsible for maintaining constant variation in populations grown from a single colony. Applying this tool on other bacterial strains can shed light on how pathogens adjust to hostile environments by diversifying their genomes.

**Electronic supplementary material:**

The online version of this article (doi:10.1186/s13073-014-0112-4) contains supplementary material, which is available to authorized users.

## Background

**Figure 1:**
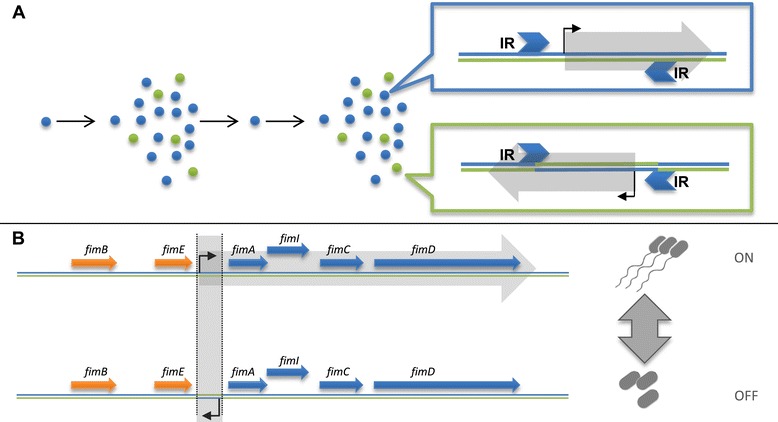
**Phase variation caused by inversion. (A)** Two genotypes (blue and green circles) are consistently and reproducibly prevalent whenever a single bacterium is grown to a population in a phenomenon termed phase variation. The two genotypes are distinguishable by a genomic inversion - a mutation which occurs when a fragment of DNA residing between two inverted repeats (IRs) is detached from the chromosome, and is then reattached in a reverse manner, resulting in a switch between the two strands. The two phenotypes may differ, for example, if a promoter located inside the fragment changes orientation and alters the transcription (gray arrow) of genes outside the inverted segment. **(B)** Phase variation in the *fim* operon. A DNA segment (shaded area) containing the *fimA* promoter can switch between two phases: an ON phase, where the promoter is correctly oriented, and the *fim* operon is expressed, and an OFF stage, where it is silenced. The OFF state also destabilizes the DNA recombinase *fimE*, probably by transcribing its antisense.

The ability of bacteria to produce heterogeneous populations has far-reaching significance in medicine and bacteriology. Over the course of evolution, bacteria have acquired complex mechanisms to produce heterogeneity within monoclonal populations [1]. These mechanisms were shown to help bacteria survive antibiotic stress [2], evade the immune system [3], and better utilize their surroundings [4]. There are several processes which, over time, can produce heterogeneity in a bacterial population. Phenotypic heterogeneity may arise from differences in the extracellular environment that may drive cells in adjacent locations toward differential activity [5]. Alternatively, bacteria can amplify stochastic processes within the cell to exhibit different gene expression profiles [6], enabling survival under stressful environments [7]. Phenotypic heterogeneity in these examples is believed to occur in genetically uniform populations. Often, lack of phenotype stability is invoked to discriminate between genetic and non-genetic contribution to phenotypic variability. However, transient phenotypic variation has been shown to occur also due to reversible genetic alterations. These alterations have to be rapid - occurring at a higher rate than typical point mutations - and reversible, thus creating two or more distinct, yet interchangeable phases. Over time, these alterations can induce the coexistence of several genotypes within the same colony. Such genotypic variation was observed long ago in a phenomenon termed phase variation (PV), where frequent genomic changes regulate the phenotypic behavior of the bacteria [8] (Figure 1A). In this work we focus our attention on variation within a population, which is derived from reversible changes in the genetic code.

Any rapidly occurring and reversible genomic alteration is prone to PV. Past studies revealed that different bacteria can produce genetic heterogeneity by specific mechanisms of genomic change. One such example is the tendency of *Neisseria meningitidis* to produce PV by slipped-strand mispairing [9]. Among the documented PV-producing mutations, inversions in the DNA sequence are major agents, shown to be the cause of well-studied PV in *Escherichia coli* and *Salmonella typhimurium* [10]. Inversions occur when a segment of DNA is detached from the chromosome and is subsequently reattached in a reverse manner (Figure 1A). For an inversion to occur, the inverted segment must be flanked by two inversely oriented repeats (inverted repeats (IRs)). The reason inversions are often linked with PV is their apparent reversibility: two inversion events between the same IRs restore the original sequence.

Inversions are the result of recombination processes [11], and as such are mediated by recombination mechanisms, either by the general homologous recombination mechanism [12,13] of the cell or by designated enzymes which recognize the flanking IRs as their target [10]. The rates at which inversion events occur in the cell may vary greatly and depend on several factors: the size of the inverted segment (the larger it is the lower the rate) [14], the size of the flanking IRs, their homology and the concentration and affinity of the mediating enzyme [15]. Inversion events may cause variability in the population if the forward and reverse flipping rates are relatively high (several orders of magnitude higher than the random mutation rate). These rates also dictate the relative abundance of each variant in the population at steady state. In the simple two variants case, the forward:reverse variants ratio is inversely proportional to that of the forward and reverse flipping rates [16].

The most studied PV in *E. coli* is the *fim* operon, which controls the expression of type I fimbriae. Coding for a surface appendage essential for interacting with host cells, *fimA* is also a major antigenic target for the immune system [17]. Clonal variation in its expression can be viewed as an evolutionary approach of bet-hedging - a risk managing strategy ensuring the survival of a subpopulation from the host’s immune response [18]. An invertible sequence of 296 bp, containing a promoter, controls the expression of the *fimA* gene, serving as an ON/OFF switch (Figure 1B) [19]. The inversion is mediated by the neighboring genes *fimB* and *fimE*. In addition to controlling *fimA* expression, the inversion also affects the stability of *fimE*, thus breaking the symmetry between the forward/reverse flipping rates [8].

While traditionally considered to be of little significance to cell function, it is now recognized that inversions may have phenotypic consequences. Small inversions encompassing a gene or part of an operon may change transcription direction, disrupt the amino acid sequence of a peptide, or create hybrid peptides. Large inversions displacing hundreds or even thousands of genes may either alter the gene expression profile by changing the location of genes on the replication arm (replichore) or hinder the replication process by disrupting the balance between the two replichores [20]. Large inversions, and the variability they produce, have been associated with various phenotypes, such as antibiotic resistance [21], reduced growth rate [22] and small colonies formation [23].

Early studies on bacterial variation singled out a distinguishable property (such as motility) in order to sort bacteria into subpopulations [19]; however, not all biological traits are easily distinguishable or easy to use as filtering criteria. Other studies compared the genomes of several clones of the same species [24] or of different species from the same lineage [25] in order to identify highly mutable sequences able to produce PV. However, this method overlooks variable loci that fail to fix in either orientation even inside a clone. Recent work aiming to discover PV using advanced sequencing methods was done in the pathogen *Bacteroides fragilis*, incorporating knowledge of IR locations and the presence of chimeric sequences to find inversions [26,27].

We suggest a systematic ‘tabula rasa’ approach, where genotypic variation is identified genome-wide, without *a priori* knowledge on its phenotypic effect and with no reliance on genomic features such as IRs. We present a new and simple method for detection of inversions and quantification of PV in bacteria via paired-end whole genome sequencing (WGS) technologies.

**Figure 2:**
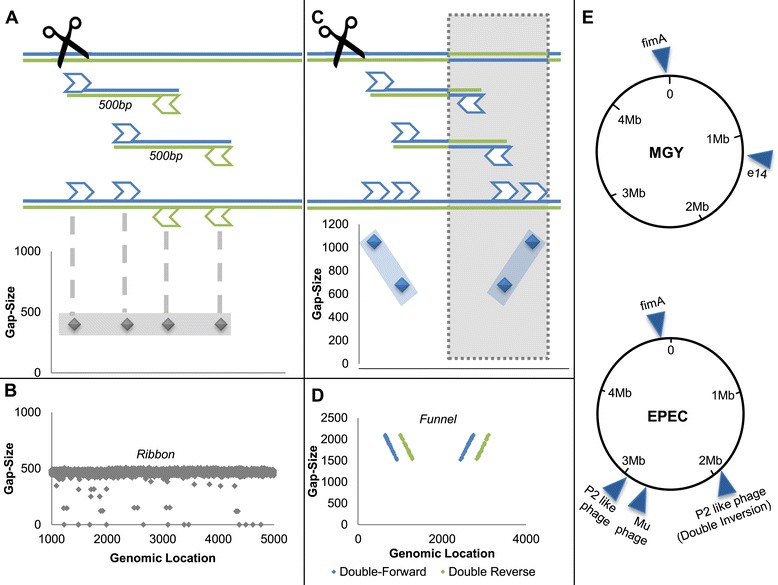
**Whole genome sequencing and detection of inversions. (A)** In the WGS process, sequenced genome is shredded into inserts approximately 500 bp long. Each insert is sequenced from both ends (paired ends), resulting in a pair of approximately 100 bp reads. Each read is mapped independently to the reference genome, and the gap size between the insert’s edges is determined for each pair. The gap size of each read is then plotted against the read’s genomic location. As long as the actual genome is identical to the reference genome, we expect a 'ribbon' formation around 500 bp (gray diamonds). **(B)** Experimental paired-end data exhibiting the ribbon formation. **(C)** When the sequenced genome deviates from the reference genome by an inversion (represented by gray shading), inserts whose reads lie on both sides of the inversion’s edge display a unique pattern that we term a 'funnel' (two symmetric diagonal lines composed of abnormally aligned reads). **(D)** Experimental paired-end data exhibiting a funnel around an inversion (blue diamonds represent plus strand paired with plus strand and green diamonds represent minus strand paired with minus strand). Note that only abnormal gap size reads are shown. **(E)** Results of the systematic inversion detection algorithm for two strains of *E. coli*. Exact genomic coordinates are available in Table S1 in Additional file 1.

Paired-end WGS produces pairs of short reads, representing the sequences of both ends of longer inserts. Since sequencing is unidirectional (from 5′ to 3′), it is normally expected that the pairs consist of one read aligned to the plus strand and another aligned to the minus strand (the complementary strand of the reference genome). It is also expected that the gap size - the calculated genomic distance between the pair - represents the original insert size (Figure 2A). These expectations combine to produce a distinct pattern, revealed when plotting read gap sizes against their genomic locations. The scattering of reads will concentrate around the actual insert size line, in a display we call a 'ribbon' (Figure 2B). Genomic areas that deviate from the ribbon pattern may indicate a genomic rearrangement.

## Methods

### Detection of inversions by paired-end whole genome sequencing

Genomic loci deviating from the reference genome by inversions display a unique pattern of paired-end WGS mapping, distinguishable from un-inverted (or normal) loci and from other chromosomal rearrangements. While plotting read gap size against genomic location normally results in a ribbon pattern composed of normally aligned pairs of reads, this pattern is disrupted by reads originating from inverted loci. Pairs of reads consisting of one read lying outside and the other read inside the inversion exhibit abnormal pairing (both reads are mapped to the plus strand or to the minus strand) and increased gap size, because the inside read changes strand orientation and genomic location due to the inversion (Figure 2C). Subsequently, plotting reads gap sizes against their genomic location reveals a unique pattern we term a 'funnel', composed of abnormal reads around inversions, replacing the horizontal 'ribbon' (Figure 2D). These two distinct characterizations of mapping, distinguishable because of the excellent quality of the WGS, allow us to scan whole genomes for inversions with a very high detection rate (Additional file 1). Once an inversion is identified, the 'inversion funnel' also allows us to examine the coexistence of the forward and reverse orientations in the population.

### Experimental setup and design

The algorithm for detection and quantification of inversions was applied on the genomes of three different strains of *E. coli*: K12 MGY (which is a derivate of the widely used commensal MG1655 expressing *yfp*), its close kin KLY, which contains the F plasmid integrated into its chromosome (*hfr*), and a well-accepted wild-type pathogenic *E. coli* (EPEC) as well as several derivates of those strains. For each strain, at least four different clones were sequenced, each clone deriving from a single colony grown on solid medium and under normal growth conditions. The growth and preparation protocols for the clones are described in Additional file 1. A summary of the PV loci detected in the sequences of all strains is presented in Table S1 in Additional file 1 and Figure 2E. Every reported PV in this paper was found to exist in similar proportions in all sequenced colonies and their existence was validated by PCR.

### Genomic extraction and whole genome sequencing

Clones were grown from a single colony to OD 0.3. Genomic DNA was extracted using QIAGEN’s DNeasy Blood and Tissue kit (from Venlo, Netherlands) Paired-end WGS was performed on Illumina HiSeq2000 by the Beijing Genomic Institute. Genomic DNA samples >6 μg (>30 ng/μl concentration) were sheared to give a mean fragment size of 500 bp. Sequencing libraries were constructed by the Beijing Genomic Institute, using a Paired‐end Sample Prep Kit. Sequencing requirements were set to an average coverage of × 100 and a read length of 90 to 100 bp. Sequencing quality was affirmed by the fastqc algorithm. Genomic analysis and manipulation were conducted in the Galaxy environment [28,29]. All WGS raw data are available as NCBI BioProject PRJNA255355.

### Mapping of clones to the reference genome

The method of creating an accurate reference genome was reported in a previous publication [30]. Sequencing data for each clone were aligned to the corresponding reference genome using the BWA alignment tool [31]. The genomic locations of reads and gap sizes of inserts were directly extracted from the mapping SAM file. The orientation of reads was calculated from the SAM bit flag data. All parts of the detection and quantification algorithm are publicly available, and a step-by-step tutorial for using the method is presented in Additional file 2.

### Mate pair sequencing

DNA was prepared similarly as for paired-end sequencing. Sequencing requirements were set to × 100 coverage and 2 kb insert size. Reads were reversed and complemented, and then aligned to the reference genome by BWA mapper similarly to PE sequencing.

### PCR validation

Each reported PV was reaffirmed using PCR. A typical PCR assay consisted of three primers, one outside the inversion boundaries and two within the inversion, such that when the outer primer was paired with each of the inner primers, it would exhibit a band.

### Sanger sequencing

The existence of micro-inversions was confirmed in the KLY mutant strain by PCR of the genomic area and Sanger sequencing from both primers.

## Results

### FimA exhibits low abundance phase variation in K12 clones

We set out to test our method on an established PV locus, *fim*, and looked for variation in it in different strains of *E. coli*. Our analysis shows PV in all sequenced colonies of MGY and KLY, albeit at low abundance. Our method not only detects the PV loci but also enables quantification of the relative abundances of the two orientations. We found that the *fim* locus is 98 to 99% in the forward orientation (corresponding to the reference genome in the K12 strains), in agreement with previous reports [8]. A similar PV was identified in the *fim* locus of the EPEC strains grown at 37°C. We conclude that our method is able to detect phase variation by DNA inversion, even when the two genotypes co-exist in relative abundances of 1:100. Analyzing the performance of our method, we conclude that at a coverage of × 100, the probability for a false negative PV at that ratio is approximately 0.04. Clearly, PVs of higher abundance have negligible rates of false negatives (see Additional file 1 for a statistical analysis).

### Reproducible phase variation of e14 prophage in MGY under standard growth conditions

**Figure 3:**
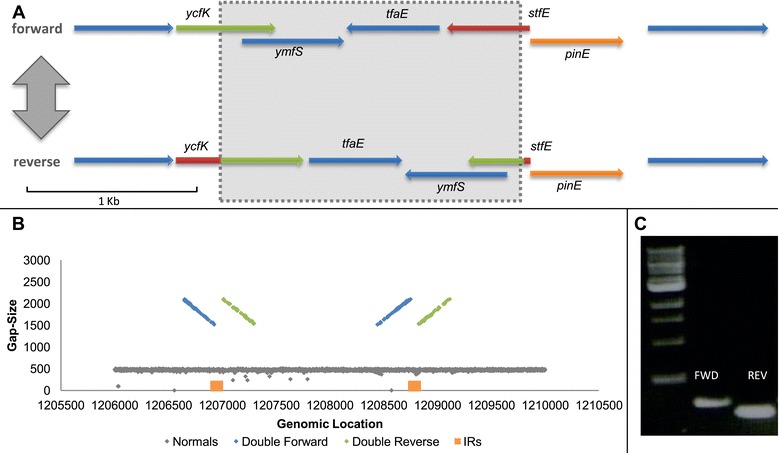
**MGY e14 phase variation. (A)** ORF analysis of the phage e14 invertible locus. The invertase *pinE* resides next to the inverted locus (represented by a shaded rectangle). In the reverse orientation *stfE* is attached to *ycfK*, producing a longer ORF than in the forward variant (fusion of the red and green segments). ORFs in all figures were inferred using SnapGene® software (from GSL Biotech, Chicago, IL, USA). **(B)** Gap size distribution plotted against chromosomal position, centered on the e14 invertible locus. Two formations coexist at the same locus: a ribbon formation of normal reads (gray), and a funnel formation of abnormal reads (blue and green). The relative abundance of each formation represents the relative fraction of each genotype in the bacterial population. The IRs flanking the inversion are marked by orange rectangles **(C)** PCR confirmation of the coexistence of two genotypes. PCR was conducted on a single MGY colony with two sets of primers. Extracted genomic DNA was used as template for both sets (see Additional file 1 for description of primers). Each band represents the existence of one orientation in the population.

K12 MG1655 is the most commonly studied lab strain of *E. coli*, and considered a model for studying bacteria [32]. We performed WGS on its derivate MGY [2]. Whole-genome search for inversions in clonal populations of MGY grown under standard conditions revealed one locus exhibiting clear PV by inversion. The inverted locus resides inside a remnant of a defective prophage known as e14 [33]. This prophage is known to harbor an invertase gene, *pinE*, which regulates the inversion of a neighboring invertible segment. An inversion event causes the fusion of two ORFs in the prophage, and might also turn on the expression of two proteins residing inside the inverted segment (Figure 3A) [33,34]. Mapping at this locus in all clones showed the co-existence of both a funnel and a ribbon formation (Figure 3B), suggesting PV. The coexistence of the two genotypes was then confirmed using PCR (Figure 3C).

Under the assumption that each WGS insert is sampled independently from the bacterial population - hence, the composition of reads represents that of the population - we discovered that the normal:flipped genotype ratio is 1:1, which is expected of a PV at equilibrium where the forward and reverse flipping rates are equal [16]. The coexistence of two equally abundant genotypes in MGY clonal populations, corresponding to each orientation, is noteworthy, and should be accounted for when considering phenotypic variability in this strain. No other PVs were detected in MGY grown under standard conditions.

### Systematic detection of phase variation in pathogenic *E. coli* (EPEC) reveals a total of three variable loci in prophages

*E. coli* (0127:H6) E2348/69 (abbreviated EPEC) is a pathogenic strain isolated from an infection [35]. Three invertible loci were identified on its chromosome. One, located in a Mu prophage, was confirmed as a PV, showing a slight tendency toward the forward orientation. Another PV was found inside a P2-like prophage (Figures S1 to S5 in Additional file 1).

**Figure 4:**
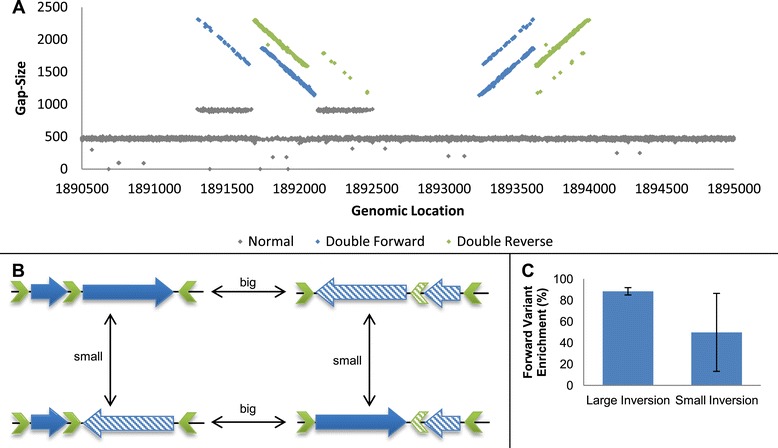
**Complex phase variation in EPEC. (A)** Two overlapping 'funnel' formations indicate a complex structure of PV. A large inversion (around 2,200 bp) and a smaller inversion (around 1,800 bp) coincide within the same module. **(B)** Sequence analysis revealed three homologous inverted repeats in the locus (green arrows), which allow for the two inversions. Further analysis indicated four possible variants. Each variant can mutate into two of the other variants by any of the two inversions. **(C)** While the large inversion retains stable proportions in all clones, the small inversion is unstable and displays great variance between samples. Error bars represent standard deviation between five independently sequenced and analyzed single colonies.

The last invertible locus found in the EPEC genome, also residing inside a P2 like prophage, showed a unique pattern of two interlaced funnels mixed with a ribbon formation (Figure 4A). We hypothesized that more than one DNA segment has the ability to undergo inversion in the locus and that more than two variants coexist in the population, a phenomenon referred to as a shufflon in the literature [36]. An analysis of the sequence identified three partially homologous IRs, which theoretically allow for two distinct inversion events to occur. We concluded that the nature of this module allows for four distinct variants (Figure 4B). Each variant can mutate into two of the other variants by an inversion event. We validated the coexistence of the four variants by PCR and, adjusting the quantification method for a four-variant case, were able to measure the abundances of each inversion event separately. Our results indicate that the big inversion remains stable between samples (where the forward variant consists of about 90% of the population), whereas the small inversion shows large variance (Figure 4C; Additional file 1).

### Detection of micro- and mega-inversions in the KLY strain

**Figure 5:**
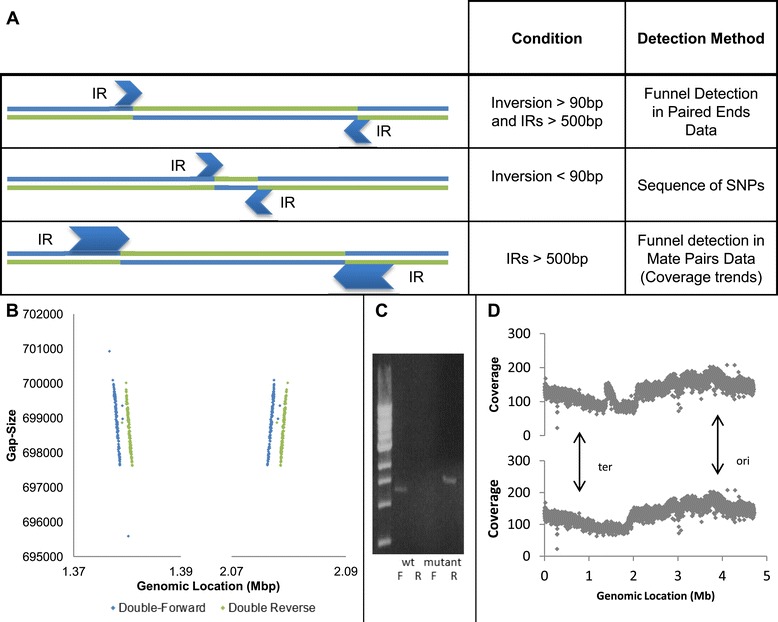
**Detection of inversions of different sizes. (A)** A summary of all inversion detection techniques presented in this paper and the conditions in which they are applicable. Small inversions will be evident as a sequence of SNPs or by a concentration of soft trimmed reads, while large inversions flanked by oversized IRs can be discovered by mate-pair WGS or by coverage trends. **(B)** Funnel detection in mate-pair data: gap size against genomic location plots centered on both ends of a mega-inversion. Mate-pair WGS with 2 kbp insert size reveals a funnel pattern in the boundaries of a suspected inverted segment. This funnel is not seen when using a 500 bp insert size. **(C)** PCR confirmation of the inversion. The wild-type (wt) and mutated strains were compared, using two sets of primers forward (F) and reverse (R), corresponding to both orientations. **(D)** Inversion detection by coverage trends. Coverage plots of the entire chromosome of the KLY mutant depict the average coverage of a genomic area against its location. Top: mapping to the reference genome reveals a 700 kbp disruption in the coverage trend caused by the mega-inversion. Bottom: mapping to a revised reference genome incorporating the mega-inversion negates the disruption. The origin of replication (ori) and replication terminus (ter) are indicated by arrows.

The 'inversion funnel' detection method relies on the existence of pairs of reads composed of one read within the inversion’s boundaries and one read outside. Inversions whose nature does not allow the existence of such pairs are thus virtually undetectable by the presented method. We extended our methodology to include the detection of such inversions as well, using WGS (Figure 5A).

### Non-variable micro-inversion in an evolved strain confers antibiotic tolerance

We sequenced six mutant clones derived from the *E. coli* KLY strain and systematically searched for inversions. All six sequenced KLY clones were isolated in a related study, where bacterial cultures were evolved under cyclic antibiotic pressure for different time intervals. These clones exhibit a distinct phenotype of increased tolerance to bactericidal treatment by significantly extending their lag phase [30]. We reported that one of the KLY derivates harbored an inversion 24 bp long, flanked by 8 bp IRs on each end. This inversion, too small to encompass a WGS read, falsely appeared as a sequence of single nucleotide substitutions in close proximity. Manual scrutiny of the mutated area revealed its true nature. Unlike other inversions reported in this paper, the KLY mutant was not heterogeneous in that locus - 100% of reads mapped to that area showed the inversion thumbprint. This inversion, whose existence was confirmed by PCR and Sanger sequencing, is located inside the F plasmid (incorporated into the bacterial chromosome), disrupting the amino acid sequence of the product of an antitoxin gene, and thus conferring a distinct phenotype of antibiotic tolerance (termed the *tbl* phenotype), as was previously reported in toxin-antitoxin mutants [37]. This observation was confirmed by genetic manipulation: deletion of the entire toxin/antitoxin module cancelled the tolerance and the wild-type phenotype was restored.

### Non-variable mega-inversion in a strain evolved under cyclic antibiotic exposure

The same KLY strain that acquired tolerance by micro-inversion after cyclic exposure to antibiotic stress was found to also harbor an inversion of approximately 700 kb, flanked by IRs of approximately 1,000 bp. Such an inversion is difficult to detect by the technique described above because of the limitation imposed by large IRs. The larger the IRs, the fewer pairs where one read is within and the other is outside the inversion. If the IR size exceeds that of the insert size, we expect no such pairs at all, making such inversions invisible to our detection algorithm.

Two complementary approaches can be combined to allow detection of inversions flanked by large IRs. The straightforward approach is to increase insert size. Indeed, by applying mate-pair WGS, with insert size averaging 2 kb, on the same mutant strain harboring a micro-inversion, we were able to detect an otherwise hidden inversion funnel (Figure 5B). The newly revealed inversion, spanning approximately 700 kb, was found to be flanked by two inversely oriented copies of the 1 kbp long insertion element *insH*. Applying the same pipeline to the mate-pair data (with relevant adjustments), we determined that the inversion is homogenous and dominates the entire population.

In addition to confirmation by a PCR assay (Figure 5C), the existence of the inversion was confirmed by examining coverage trends in regular paired-end WGS of the same strain. Bacteria sequenced at the exponential growth phase show a significant decreasing trend in read coverage between the origin of replication and the terminus, due to ongoing parallel replication of DNA at the origin of replication. When this trend is non-monotonic, it might indicate that a large chromosomal rearrangement has occurred between the sequenced clone and the reference genome [38]. Coverage trend plots of the mutant strain show a clear disruption in the area of inversion, while mapping the strain to a reference genome incorporating the inversion makes the disruption disappear (Figure 5D). This finding supports our detection of the inversion by funnel detection in mate-pair data, and shows that paired-end WGS can sometimes be applied to discover inversions whose IR size exceeds the insert size.

The inversion was subsequently characterized by means of a conjugation assay, transferring the inverted locus as a whole to a different strain. PCR and WGS were then applied to the recipient strain to confirm the presence of the inversion. The recipient strain showed no phenotypic difference from the wild type, establishing that the inversion had no apparent effect on phenotype. The conjugation protocol and the analysis of the recipient strain are depicted in Additional file 1.

## Discussion

We present a simple method for detection and analysis of genetic variation in bacterial populations. Our method is based on WGS data and relies on the misalignment of reads inside inverted loci as indicators of inversion events. We show that under the sequencing scheme used here, it can detect inversions that occur in only 1% of the sequenced population with a low rate of false negatives (<5%). We also suggest complementary ways for the detection of inversions whose nature prevents detection by our methods.

The same pipeline is applicable both to paired-end and to mate-pair technologies, and with modest tweaking can cover a wide range of genomic alterations. Genomic variation and PV can be caused by agents other than inversions: slipped strand mispairing [39], insertion/excision [40] or amplification/deletion [41] to name a few examples. Since all of these genomic alterations leave a distinct and recognizable signature on WGS mapping, detection and quantitative analysis of PV caused by these alterations is feasible using very similar methods, and might be used to better comprehend the inherent genetic variability in seemingly clonal bacterial populations. Similar methods can also be used to characterize diversity in batch cultures, keeping track of emergence and fixation of genomic rearrangements [42].

Several limitations of our method should be mentioned. The existence of the inversion funnel depends on WGS parameters, specifically read and insert sizes and coverage depth. Detection of inversions which do not display the funnel requires altering these parameters (for example, increasing insert size) or applying complementary approaches (for example, coverage trends). Another inherent shortcoming of our method is lack of external validation for the quantitative aspect. An encouraging finding is the detection of the mega-inversion both by coverage trends and by mate-pair sequencing.

Applying our methodology on widely used strains of *E. coli*, we demonstrate that these strains constantly produce heterogeneous populations, in a predictable and reproducible manner. Apart from the *fim* textbook case, all variable loci detected are within prophages and were previously identified as segments that might be found in different orientations in different strains [34,43,44]. These loci are recognized by enzymes which are close homologs of the *hin* gene responsible for PV in *Salmonella* [45]. Our findings indicate that, in standard conditions, these segments constantly flip, producing two or more genetically distinct subpopulations within the same culture originating from a single colony.

Two processes can equally account for the observed phenomena: either enough flipping events had occurred to reach equilibrium by the time DNA was extracted from the population; or the genotype of the founder bacterium is still dominant and is slowly decaying. In order to resolve which of these hypotheses is correct, we need a good estimate of the number of divisions and of the absolute flipping rates. The number of divisions required to form a colony from a single cell on LB agar is estimated at 10^9^. Additional growth on liquid LB prior to DNA extraction results in approximately 2 × 10^9^ divisions. Flipping rates are hard to estimate, and can vary widely, which means that each PV should be judged separately. Flipping rates for *fimA* in MG1655 were previously estimated at 10^-3^ and 10^-1^ events per division for OFF → ON and ON → OFF transitions, respectively [8,19]. Our findings that the forward:reverse proportions were approximately 100:1 agree with the hypothesis that the variants are at equilibrium. Solving a dynamic model of the inversion with the estimated parameters of *fimA* confirms that the population reaches steady state long before DNA extraction (Additional file 1).

The same basic variation mechanism - the combination of an invertase and a set of IRs - can produce complex processes. We found a set of three IRs whose positioning allows for four different genomic variants and three alternative carboxyl termini for the same protein, thus broadening the range of available phenotypes. We found that all four variants coexist in the population. A simpler version of the same mechanism (in a different P2 like prophage) produces only two variants. Thus, the architecture of IRs plays a major role in variation production.

The phenotypic effect of the PV reported in this work is yet to be fully understood. All variable sequences found in phages are used by the phages to alternate between tail fiber structures [35], in order to diversify their host range specificity [46] as a bet-hedging strategy that increases chances of survival after lysis [47]. However, over the course of evolution bacteria can assimilate prophages and use their genetic material for their own benefit [48] and it is intriguing to speculate whether our investigated strains utilized these inherent heterogeneity-generating processes for other purposes of medical significance. For example, a recent study demonstrated how the commensal *Xenorhabdus bovienii* utilizes P2-type prophages to compete with other bacteria in its environment, potentially channeling the phage’s host-range diversity to its own advantage [49]. This utility of prophage heterogeneity might have a role in shaping the composition of the microbiome and combating pathogenic invasions. Additionally, the conservation of these invertible sequences in many bacterial strains also suggests an adaptive role in bacterial evolution [50]. We also report two homogenous inversions in a mutant of KLY evolved under antibiotic stress, dominating the entire population. Of these two, the micro-inversion was shown to have a phenotypic effect of increased tolerance to antibiotic, whereas the mega-inversion was found to have no effect on cell behavior (Additional file 1). It would be interesting to investigate further whether antibiotic exposure itself can promote the appearance of inversions of various sizes.

The term ‘phenotypic variability’ is often used to describe the phenomenon where two cells behave differently although they contain identical genetic content [1]. However, the evidence for identical genetic content is usually inferred from the fact that the culture originated from a single colony, and that the phenotypic variability is maintained through re-growth after inoculation of any of the subpopulations. Considering the prevalence of PV presented in this paper, accepted cases of phenotypic variability could theoretically be caused by hidden genetic mutations. Therefore, we sequenced an *E. coli* KLY strain containing the *hipA7* mutation, which causes an increase in the number of persister (or dormant) cells in the population (10 to 30%), thus inducing greater population variability [37]. This mutation was previously connected to the threshold-based amplification of gene expression noise [7]. We used our methodology to test whether a PV-related mechanism could be detected and conducted WGS mapping to search for variable loci. No genotypic variation was found in that strain, substantially supporting the understanding that the phenotypic heterogeneity observed in this strain is indeed non-genetic.

The emergence of next generation sequencing heralded a revolution in the ability to comprehend the entirety of genomic processes. At first, researchers were content to apply this technology for the discovery of point mutations. Later, genomic rearrangement discovery techniques were developed [51]. We view the analysis of inherently variable sites as an important tier in this shared effort.

## Conclusions

By using simple computational tools we demonstrate how genetic heterogeneity caused by inversions can be identified, measured and modeled. We show that commensal and pathogenic strains of *E. coli* use inversions as mechanisms for producing genetic heterogeneity. While the function of this mechanism remains to be fully resolved, it is clear that genetic heterogeneity can contribute to fitness, especially for pathogens which must perform various tasks simultaneously in hostile environments. An array of sequencing techniques and detection tools can be combined in order to attain a complete picture of the diversity of genomes in seemingly clonal bacterial populations.

## Additional files

Additional file 1:
**Table S1, Figures S1 to S7, and Supplementary text and methods.**
Additional file 2:
**A tutorial for detection and quantification of inversions.**

## Abbreviations

bp: base pair
IR: inverted repeat
ORF: open reading frame
PCR: polymerase chain reaction
PV: phase variation
SNP: single-nucleotide polymorphism
WGS: whole genome sequencing
