# Supplementary material for: Systematic identification and quantification of phase variation in commensal and pathogenic Escherichia coli
Source: Genome Med. 2014 Nov 28;6(11):112. doi: 10.1186/s13073-014-0112-4 (PMC4272514; doi:10.1186/s13073-014-0112-4)
Supplement: Additional file 1: — Table S1, Figures S1 to S7, and Supplementary text and methods. [file 13073_2014_112_MOESM1_ESM.pdf]

Table S1: Detected PV events in all sequenced strains

Supplementary Text

1. **PVs inside mu and P2 prophages in EPEC**
2. **Theoretical Analysis of Heterogeneity Producing Mechanisms**
3. **Four States Variation - Model Construction and Solution**
4. **Transmission of Mega Inversion to a New Strain via Conjugation**
5. **Supplementary Methods**

Strain	NCBI Accession Code	Start Location	End Location	Locus/ Prophage	Number of sequenced colonies	Avg. forward %	Inverted Repeat Sequence
MGY and derivates	MG1655 (U00096.3)	4542683	4542995	*fim*[*]	4	99 ± 1	TTGGGGCCA
MGY and derivates	MG1655 (U00096.3)	1207008	1208846	*e14*	4	51 ± 6	AAACCTTGGTTTGGGAGAAGG
EPEC	0127:H6 E2348/69 (NC_011601.1)	2749478	2751398	*Mu*	4	60 ± 8	AAACCTTGGTTTGGGAGAA
EPEC	0127:H6 E2348/69 (NC_011601.1)	1891695	1893703	*P2*	4	90± 3	**
EPEC	0127:H6 E2348/69 (NC_011601.1)	1892127	1893703	*P2*	4	49 ± 37	**
EPEC	0127:H6 E2348/69 (NC_011601.1)	2977580	2979467	*P2*	4	93 ±4	TTGGTTTGGGAGAAG

Table S1: Detected PV events in all sequenced strains

[*] Note that in some, but not all, of the EPEC clones a PV in the *fim* locus was detected, and therefore it is not listed here

[**] The IR sequences responsible for the double inversion are 215 bp each and show 92% homology. The leftmost IR sequence is:

ACCTTGGTTTGGGGGAAGGCTCTGCACTGCCCGTTGGTGTGCCCGTTCCGTGGTC
CTCAGCAACACCACCAACGGGCTGGCTGAAATGTAACGGTGCAGCATTTTCTTCT
GAAATGTATCCCAGACTGGCAAGGGCTTATCCCACCAATAAATTACCGGATTTAC
GCGGTGAATTTATCCGTGGCTGGGATGATGGGCGCGGGATTGATGCGGGA

1. PVs inside mu and P2 prophages in EPEC

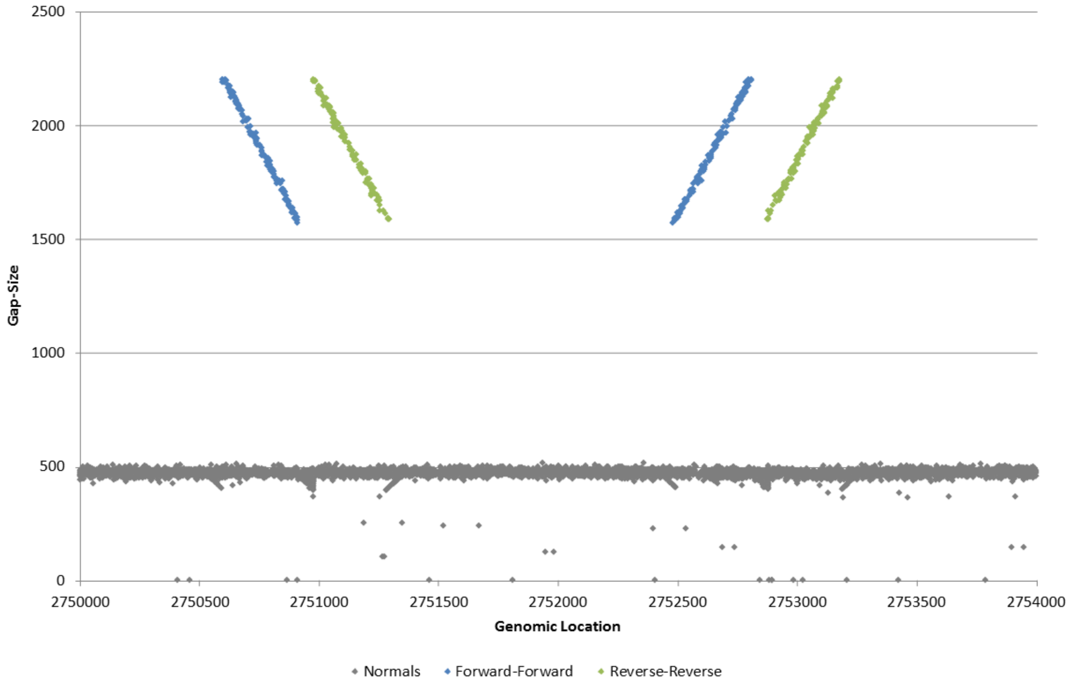

Figure S1 - Phase Variation in EPEC: mu Phage

Gap-size against genomic location plot around the invertible locus in the mu phage. The presence of both the inverted (funnel) and non-inverted (ribbon) genotype can be seen.

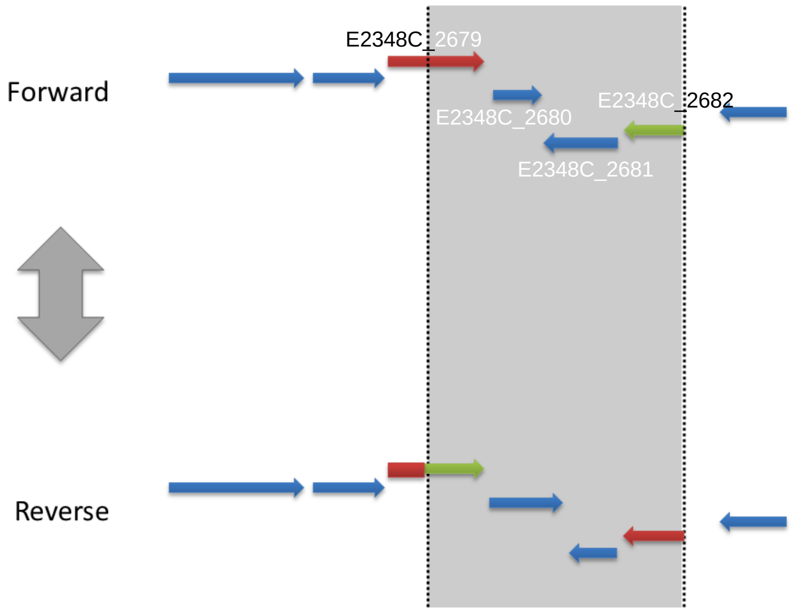

Figure S2 - ORF Analysis of mu Phage Invertible Locus

Inversion causes fusion of alternative C terminus to the protein upstream of the inverted segment

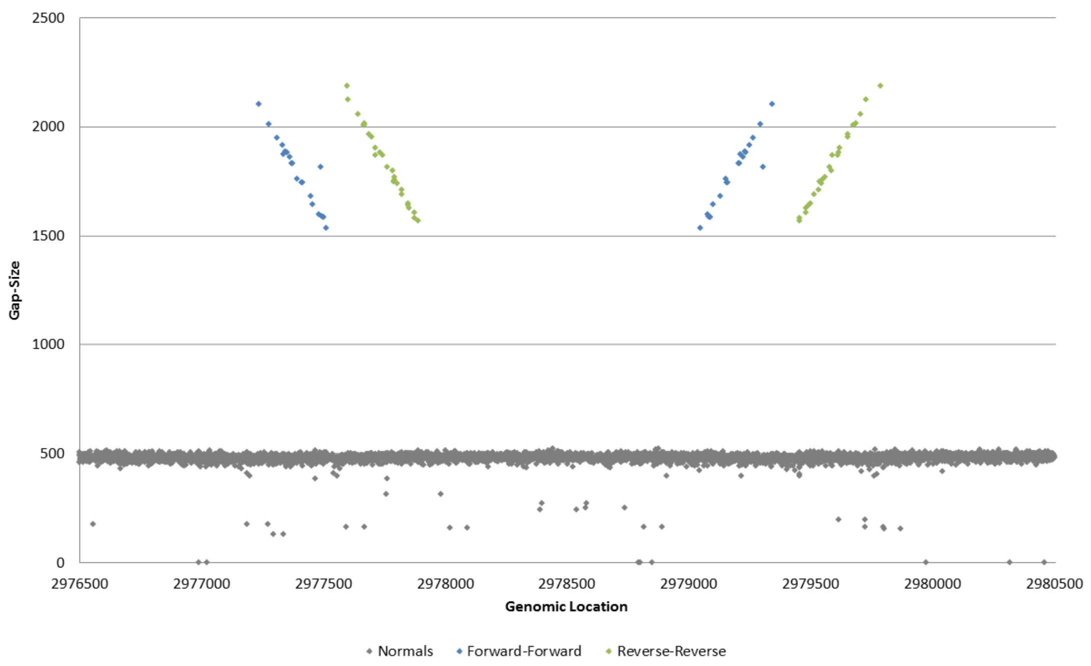

Figure S3- Phase Variation in EPEC: P2 Phage

Gap-size against genomic location plot around the invertible locus in the mu phage

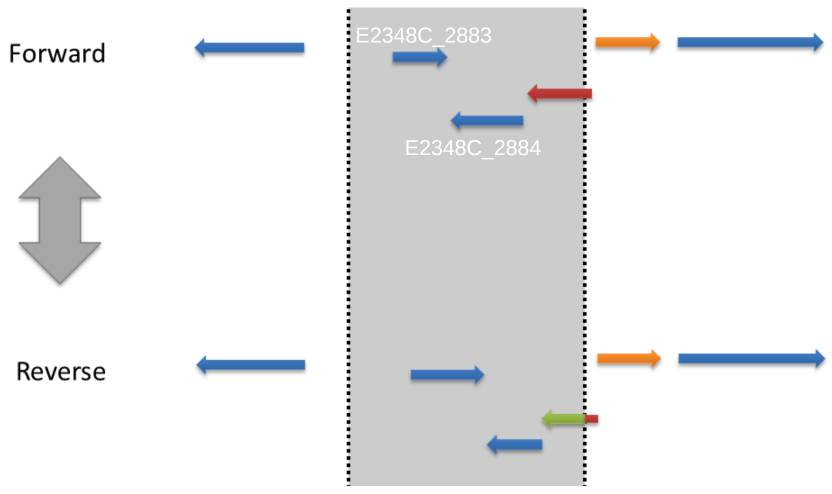

Figure S4 - ORF Analysis of P2 Invertivle Locus

Each orientation attaches a different C-terminus to a protein downstream of the invertible locus

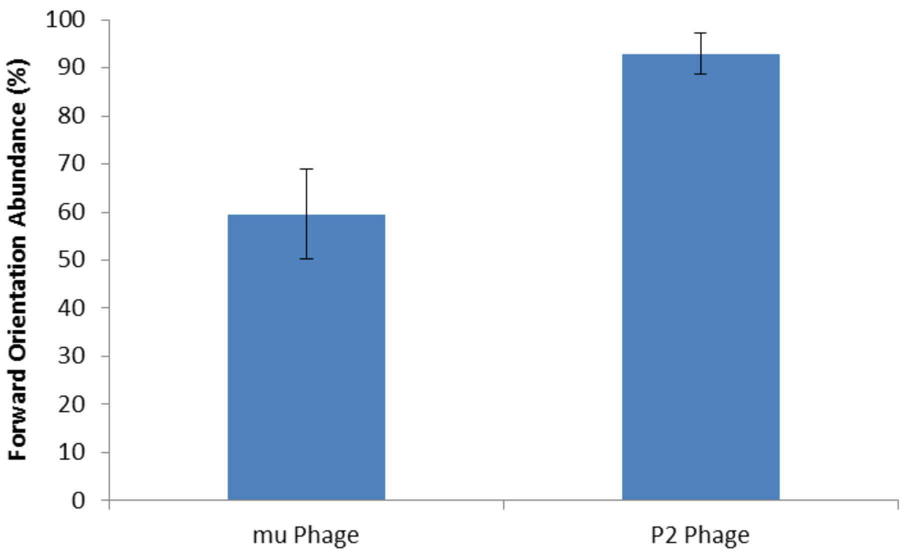

Figure S5 - Abundance of Forward Orienation in EPEC PVs

Error bars represent standard deviation between samples.

2. Theoretical Analysis of Heterogeneity Producing Mechanisms

In this analysis we assume that growth rate is equal in both variants. A general solution for a two-state growth problem, where growth rate of the two states is not identical appeared in an earlier publication [1].

To estimate just how high flipping rates must be to produce population variability, let's consider the simple case where forward and reverse flipping rates are equal (α), and we start with a single bacterium of the forward orientation (variant A). At equilibrium, variants A and B should be at equal proportions in the population. The equation that describes the progression of B toward that equilibrium concentration is:

Equation 1:

$$c = \frac{B}{A+B} = \frac{1}{2}\left[1 - e^{-2\alpha t}\right]$$

where α represent flipping rate and t represents time. From this equation we can conclude that for B to dominate the c fraction of the population by the time t, α should be greater than:

Equation 2:

$$\alpha \geq -\frac{\log(1-2c)}{2t}$$

For example, to reach 10% abundance of the reverse orientation by the time population reaches 10^9 cells (standard number of bacteria in a colony), flipping rates must be greater than 0.5% of the standard growth rate, much higher than random mutation.

In the case where the forward flipping rate (α) differs from the reverse rate (β), the ratio of the variant B is determined by the equation:

$$\frac{B}{A+B} = \frac{\alpha}{\alpha+\beta}\left[1 - e^{-(\alpha+\beta)t}\right]$$

From this equation we conclude that the timescale for the system to reach steady state is $\tau = (\alpha + \beta)^{-1}$. Now let's consider the fim locus case in MG1655. Previous studies estimated the flipping rates to be: $\alpha_{fim} = 10^{-1}$ divisions^{-1} $\beta_{fim} = 10^{-3}$ divisions^{-1} [2]. Hence, τ_{fim} = 10 divisions. Since we grow the bacteria from one cell to a population of

$\sim 10^9$, we can conclude that the passing time $t = 10^9$ divisions $\gg \tau_{fim}$ and that our sequenced clones are at equilibrium in the *fim* locus.

3. Four States Variation - Model Construction and Solution

In order to understand the dynamic behind the complex Phase Variation, we devised a simple model containing the fours variants and their transition probabilities.

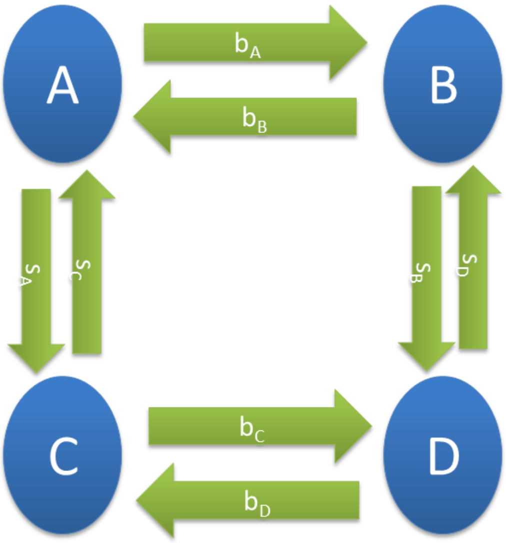

Figure S6 - Four Variants Model

The simple model assumes equal growth rate (μ) for all variants. Each transition between a state to another (which signifies a flipping event) is assigned a probability $b/s_{ABCD,}$ where b/s signifies the big or small inversion respectively, and ABCD represent the exit state's denomination (for instance, s_A represents a small inversion event from state A to state C). The equations stemming from the model are:

Equation 3

$$\frac{dA}{dt} = (\mu - b_A - s_A)A + b_B B + s_C C$$

$$\frac{dB}{dt} = (\mu - b_B - s_B)B + b_A A + s_D D$$

$$\frac{dC}{dt} = (\mu - b_C - s_C)C + b_D D + s_A A$$

$$\frac{dD}{dt} = (\mu - b_D - s_D)D + b_C C + s_B B$$

With no other simplifying assumption, solving the equations for the relative abundances of each state (i.e. A/N_t) for steady state reveals that only one stable solution exists:

Equation 4

$$\frac{A}{D} = \frac{b_B \cdot b_C \cdot s_D + b_B \cdot b_D \cdot s_C + b_B \cdot s_C \cdot s_D + b_D \cdot s_B \cdot s_C}{b_A \cdot b_C \cdot s_B + b_B \cdot b_C \cdot s_A + b_A \cdot s_B \cdot s_C + b_C \cdot s_A \cdot s_B}$$

One possible simplifying assumption is that flipping rates of the same inversion remain constant, regardless of the exit state. In that case, we add the constraints: $b_{A=}b_C$ $b_{B=}b_D$ $s_{A=}s_B$ $s_{C=}s_D$, and get:

Equation 5

$$\frac{A}{D} = \frac{b_B \cdot s_C}{b_A \cdot s_A} \qquad A = \frac{B \cdot C}{D}$$

This assumption is not supported by our data, where A deviates from (B*C)/D by a factor of 2.

This model describes the four-state inversion at equilibrium. Since we have strong evidence that this system has not reached steady state (great fluctuations in the small inversion between samples), we conclude that it cannot account for the observed abundances.

4. Transmission of Mega Inversion to a New Strain via Conjugation

Following the discovery of the emergence of a 700 Kbp mega inversion in an E. coli KL16 mutant, we set out to characterize both the dynamics and the phenotypic effect of the inversion. For that purpose we transferred the inversion to a new wt strain via conjugation.

Since the size of the inversion barred the possibility of using standard genetic engineering methods, we exploited the fact that our strain is *hfr* [3], hence can be used as a donor of its chromosome in a conjugation procedure. The term conjugation refers to the biological process where a bacterium transfers its fertility plasmid to another bacterium. An *hfr* bacterium, having the plasmid incorporated to its chromosome, can advertently transfer part of its chromosome along, which can then merge into the recipient's chromosome by means of homologous recombination. The conjugation always starts at the same point and in the same direction, dictating a certain order by which the genomic regions are transferred to the recipient. It is important to remember that only part of the linear segment will eventually be incorporated into recipient's chromosome and inherited to the next generations, and that it is determined randomly during the recombination process [4].

Our goal was to isolate conjugants that incorporated the large inversion (preferably with as little additional DNA content as possible) into their genome. For that purpose, we used P1 transduction to add two antibiotic markers to the mutant strain, flanking the inversion from both sides (we named this new strain KLYC). As the recipient strain we chose a member of the Keio Collection [5] containing an antibiotic marker embedded in its chromosome at a location of our wish. The complete conjugation protocol is depicted in the Supplementary Methods section. In short, both donor and recipient strains we mixed together in LB and incubated at 37°C with no shaking for 2 hours. Afterwards, the mix was diluted and plated on restrictive media in order to isolate conjugant strains.

We isolated several conjugants clones, and genotyped them by PCR assays. As was expected, some isolates did not transfer the inversion while a few did. We compared the growth dynamics of the isolates with and without the inversion to conjugant isolates from the control assay and to the original strains. We found no phenotypic difference between the conjugant and its counterparts. We conducted Paired-End

WGS on the conjugant. Coverage trend showed clearly that the inversion transferred as a whole (Figure 20). We were also able to get a good estimation on conjugation boundaries, by examining genomic differences (SNPs) between the donor and recipient.

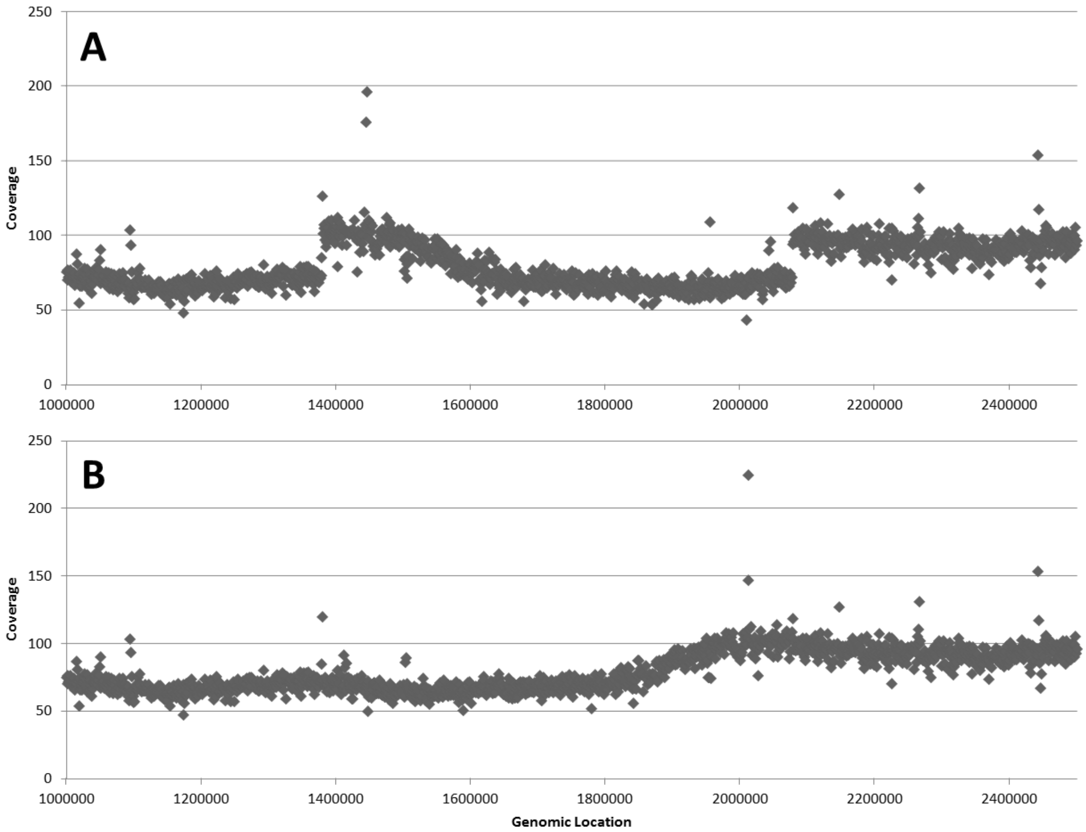

Figure S7 - Mega Inversion Transmission Through Conjugation

A. Coverage plot of the conjugant shows the clear signature of the inversion, and indicates that the inversion was transferred as a whole to the recipient strain. **B.** By aligning the conjugant's WGS data to a reference genome incorporating the inversion, the inversion signature disappears.

5. **Supplementary Methods**

Detection of Clusters of Abnormal Reads

Inversion detection algorithm works as follows. After mapping the reads to a reference genome corrected for genomic rearrangements and SNPs, abnormal reads of forward-forward pairings (where both reads are on the plus strand) are identified.

The algorithm randomly picks a read. It then defines a geometric shape around the read in the plane where the x axis represents genomic location and y axis represents gap-size. The algorithm finds all abnormal reads whose genomic location is adjacent to the read and which are positioned on top of the straight line whose slope equals -2 that intersects the pivot read (with predefined error margins) and scores the read's genomic location for the abundance of such reads. It then removes the entire cluster from the group of reads, and continues to the next iteration until the group is empty. High scoring clusters are identified as suspected inversions, and undergo manual inspection and PCR validation.

Theoretical analysis shows that if there was no noise, all funnel reads should be aligned on a -2 sloped line. Failure to detect an inversion (a false negatives) should stem from two main reasons: Either the reverse orientation abundance is low, so that Binomial distribution properties sets the number of abnormal reads below detection threshold, or there are enough abnormal reads, but gap-size noise sets a few outside detection boundaries.

To determine the probability that a real inversion event won't be detected by our algorithm, we defined an event A = [our algorithm fails to detect a cluster of more than 3 reads]. We also defined several parameters:

a – the error margins used in the algorithm

cov – the total number of reads in the algorithm's window size. Its distribution can be empirically inferred

x – the deviation of the selected read from the gap size distribution. We assume gap-size is distributed normally.

reads – the number of abnormal reads from the inverted population

We calculated the probability P(A) for inversions with different enrichment values p%.

Equation 6

$$P(A) = \sum_{cov,x,reads} P(A|cov,x,reads) \cdot P(cov,x,reads)$$
$$= \sum_{cov,x,reads} P(A|cov,x,reads) \cdot P(x) \cdot P(cov) \cdot P(reads|cov)$$

Now, if reads is lower than 3, P(A|cov,x,reads)=0, otherwise:

Equation 7

$$\boldsymbol{P(A|cov,x,reads) = [P(y < x - a) + P(y > x + a)]^{reads-2}}$$

We assume that P(reads|cov)~Binomial(cov,p) and that P(x) is a normal distribution (whose parameters are inferred empirically). We found that P(A) is $3{\cdot}10^{-5}$ per sequenced clone for p=5%, but is 4% for p = 1%.

Another important factor affecting the efficiency of our detection algorithm is sequencing and mapping quality. Since our algorithm relies on pairs of reads, it can only use inserts where both ends were mapped to the reference genome. If, for example, 5% of the reads were corrupted, only 90% of the reads are usable, affecting the effective coverage at each site.

Construction of inverted reference genome

Once an invertible locus was identified, its genomic variants are carefully deciphered. This is done by isolating soft-trimmed reads: reads only partially mapped to the reference genome, with an overhanging unmapped residual tail. While soft trimming may be caused by many reasons (such as sequencing cumulative errors), an invertible locus is typically enriched with soft trimmed reads stemming from the inverted sub-population. Soft-trimmed reads are used to tailor the exact sequence of the inverted variants and for the construction of alternative reference genomes.

Quantification of Inversion

Each clone is mapped to truncated reference genomes, containing only the variable locus in a different orientation each time. Variable reads are identified and counted. We term a read as variable if it maps normally to at least one orientation of the locus and abnormally to others. Abnormal mapping to a reference genome is either of the following:

Abnormal pairing (forward-forward, reverse-reverse)

gap-size surpasses the normal distribution of segment sizes

The read is soft-trimmed

In a two-phase locus, the composition of variable reads, mapping correctly to one orientation but not to the other, is proportionate to that of variants in the whole population.

In the four-phase locus found in EPEC, inferring the exact composition of the population is more complicated, since variable reads can map correctly to more than on reference, so it is not always clear exactly which variant the read originated from. To overcome this obstacle we divided the variable reads to several groups. A variable read whose pair lie to the "left" of the inversion, for instance, can belong to one of three mutually exclusive categories: Either it maps correctly to variant A (category A) exclusively, to variant C (category C) or to variants B and D (category BD). Thus, the relative abundance of variant C in the whole population is proportionate to category C's size in relation to the sum of all three categories.

Strains

All MGY clones are products of P1vir of intC::YFP-CAM from MRR into MG1655. MRR was kindly provided by Michael Elowitz [6]. 3 of the used MGY clones are products of P1vir of *ΔmutS*-KAN from the KEIO collection into MGY [5]. Note that deletion of *mutS* in these clones does not alter PV experimental ratios.

All KLY clones are products of P1vir of intC::YFP-CAM from MRR into KL16. MRR was provided by Michael Elowitz. KL16 was kindly provided by Hooper [7]. KLY reference genome was published in a previous study (Accession No. CP008801)

E2348/69 EPEC bacteria were kindly provided by Ilan Rosenshine. Each clone was diluted from starter culture or directely from colony into the fresh LB medium. These cultures were grown at different temperatures to get FimA 28°C, 32°C and 37°C to get different *fimA* switching. Bacteria were collected at O.D.=0.35.

The KLYC strains used for the conjugation assay is a product of P1vir of galK::CFP-AMP from MRR into both KLY (control) and the mutated strain of KLY.

The strain used as recipient in the conjugation assay is BW25113ΔlacY, obtained from the KEIO collection, having its lacY locus replaced with a kanamycin resistance cassette by P1 transduction [5].

Conjugation Protocol

Three strains were used in this experiment:

Recipient: BW25113ΔlacY (resistant to kanamycin (KAN))

Donor: KL16 yfp-CAM cfp-AMP with mega-inversion (resistant to chloramphenicol (CAM) & ampicillin (AMP))

Donor: KL16 yfp-CAM cfp-AMP (control, resistant to CAM & AMP)

All strains were plated and grown from single colony overnight. Strains were diluted and grown to the same OD (~0.4). 0.2 ml of donor strain were mixed with 0.2 ml of recipient and incubated with no shaking at 37° for two hours. Mixture was then vortexed thoroughly to stop all active conjugation. 0.2 ml LB was added to the mixture. Mixture was incubated with shaking at 37°C for two hours.

The mixture was plated with different dilutions (1:100 to 1:10^5) on different media:

LB + 12.5 μg/ml CAM+ 100 μg/ml AMP + 30 μg/ml KAN (only conjugants should grow here).

LB + 12.5 μg/ml CAM + 100 μg/ml AMP (recipients + conjugants should grow here).

LB + 30 μg/ml KAN (donors + conjugants should grow here).

Colonies were counted for calculating conjugation efficiency.

Selected conjugant colonies were isolated, grown and tested by PCR for inversion transfer and antibiotic tolerance. One conjugant colony positive for the inversion was then processed for WGS.

Used PCR Primers

e14 Phase Variation

e14pv_out	GCGGCACGACCAGTTACTTA
e14pv_norm	ACGCAACCGGGAATACAACT
e14pv_inv	AAAGCGGCACCATTGCATTT

EPEC Double Inversion

dob_left	AACCGCGTTGACAAGTGTTG
dob_right	ATCCCGGTCTGGCTGATTTC
dob_fwd	TGTCATTTGGCACCAACACC
dob_rev	TGGCCAACTCCCATTCATCC

Mega Inversion PCR

MGY5HL2_LInv_OUT	TCAGGGAAGGAAGTAGCAACA
MGY5HL2_LInv_NORM	TACGTGAACCGGGTCACACT
MGY5HL2_LInv_INV	ACTCCTGTCAGGTGTGATCA

1. Balaban N, Merrin J, Chait R, Kowalik L, Leibler S: **Bacterial persistence as a phenotypic switch.** *Science (New York, NY)* 2004, **305:**1622-1625.
2. van der Woude M, Bäumler A: **Phase and antigenic variation in bacteria.** *Clinical microbiology reviews* 2004, **17:**581.

3. Ippen-Ihler K, Minkley E: **The conjugation system of F, the fertility factor of Escherichia coli.** *Annual review of genetics* 1986, **20:**593-624.
4. Smith G: **Conjugational recombination in E. coli: myths and mechanisms.** *Cell* 1991, **64:**19-27.
5. Baba T, Ara T, Hasegawa M, Takai Y, Okumura Y, Baba M, Datsenko K, Tomita M, Wanner B, Mori H: **Construction of Escherichia coli K-12 in-frame, single-gene knockout mutants: the Keio collection.** *Molecular systems biology* 2006, **2:**2006.
6. Elowitz MB, Levine AJ, Siggia ED, Swain PS: **Stochastic gene expression in a single cell.** *Science (New York, NY)* 2002, **297:**1183-1186.
7. Breines DM, Ouabdesselam S, Ng EY, Tankovic J, Shah S, Soussy CJ, Hooper DC: **Quinolone resistance locus nfxD of Escherichia coli is a mutant allele of the parE gene encoding a subunit of topoisomerase IV.** *Antimicrobial agents and chemotherapy* 1997, **41:**175-179.
