# Supplementary material for: Systematic identification and quantification of phase variation in commensal and pathogenic Escherichia coli
Source: Genome Med. 2014 Nov 28;6(11):112. doi: 10.1186/s13073-014-0112-4 (PMC4272514; doi:10.1186/s13073-014-0112-4)
Supplement: Additional file 2: — A tutorial for detection and quantification of inversions. [file 13073_2014_112_MOESM2_ESM.pdf]

A Short Tutorial for Detection and Quantification of Inversions

This tutorial's purpose is to instruct how to use Whole Genome Sequencing (WGS) data in order to detect genomic inversions in the genomes of bacteria and calculate their prevalence in the population. It is written as a supplementary guide to the paper "Systematic Identification and Quantification of Phase Variation in Commensal and Pathogenic *E.coli*". This step-to-step guide instructs how to find and quantify inversions. The MATLAB functions used in this tutorial can be downloaded from Balaban lab's website.

a. Map WGS to Reference Genome

Use BWA to align the paired ends sequencing data (two fastq files, one for forward reads and one for reverse) to the reference genome (fasta file).

The output of the BWA algorithm is a SAM file. SAM is a text tabular file where each row represents a single WGS read. Only a few of the table's fields are needed in our algorithms:

i. Identifier (c1): each WGS pair has a unique identifier. Pairing of the two reads is possible with this field
ii. Bitwise flag (c2): Contains information on the read's mapping. This field is used to extract abnormal pairing
iii. Genomic location (c4)
iv. SAM identifier (c6): Is used to identify soft-trimmed reads.
v. Gap size (c9)

Save the resulting text file for further analysis

b. **Prepare data for detection**

First the SAM file must be loaded to MATLAB. Use the command line:

```
data = read_sam_file(filename);
```

to create the matrix data, which contains 3 columns (c2, c4 and c9).

c. Use Inversion Detection Algorithm

As is described in the Supplementary Methods, the algorithm for inversions detection searches for clusters of abnormal reads concentrated on a sloped line

forming the funnel pattern when plotting gap-size against genomic location.

To execute the algorithm, run the command line:

```
[location, score] = detect_inversions(data);
```

The output of the function is a vector of genomic locations containing putative inversions and a corresponding vector of scores, based on the size of the cluster of abnormal reads in that location.

d. Visualize the Inversion (funnel or contact matrix + ref)

Once we obtain a set of putative inversions, we can visualize each genomic location and inspect the funnel pattern that it produces. Run the command line:

```
region = reads_over_region(data, center, range);
```

in order to visualize the funnel pattern around the putative inversion. Set the parameter `center` to the genomic location you wish to inspect. Running this command on the e14 inversion reported in the paper will produce the following figure:

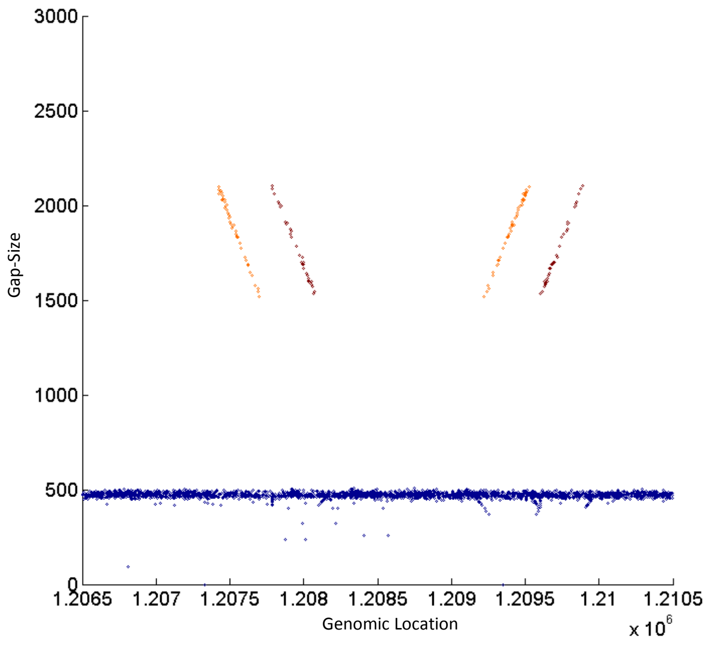

The output of the function is `region`, which is a matrix containing all reads that map to the area around the inversion. An alternative way to view the inversion is by plotting a read's location against its pair's (a contact matrix view), by running:

```
plot(region(:,2), region(:,2) + region(:,3), '.');
```

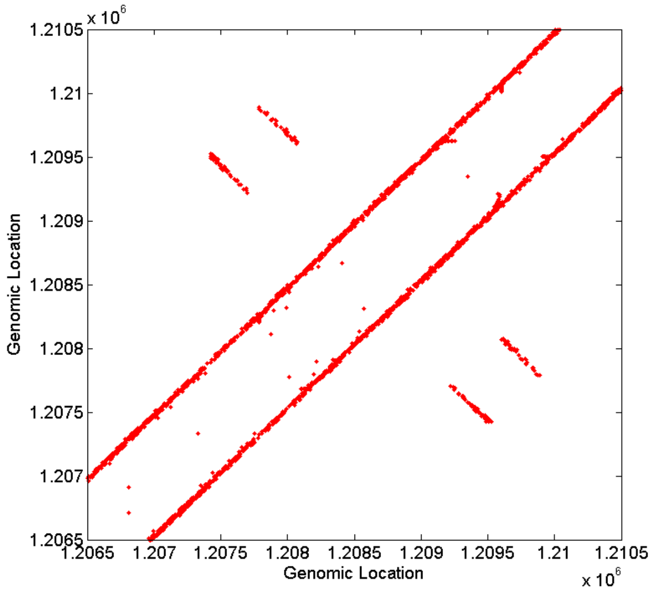

e. **Identify Inversion's Edges**

One of the ways to identify inversion's edges is to find hybrid reads – reads that combine two separate genomic sequences. BWA treats these reads by trimming them and mapping only one part to the genome. When a cluster of reads is soft trimmed at the same genomic location, it might mark the edge of an inversion. Run the command line:

```
[location, trimmed] = find_trimmed_clusters(region)
```

to get a list of all the genomic locations marked by trimmed reads' edges. Use this function to determine the inversion's edges.

f. **Prepare Two Reference Genomes**

After we identified a funnel-forming inversion, we now wish to quantify how abundant it is in the population. Our approach for quantifying an inversion is to map the WGS data to a small part of the genome with and without the inversion, single out the pool of reads that map normally to one genome and abnormally to the other (variable reads), and calculate the fraction of reads which map normally to the inverted genome of the pool of variable reads. First we want to create to small reference genomes containing the genomic area around the inversion. Run the command line:

```
prepare_reference_genomes(reference_genome, center,
range, left_boundary, right_boundary)
```

This function receives a fasta file of the reference genome, and the boundaries of the inversion and prepare two smaller fasta files containing the inverted

locus, one with the inversion embedded to the sequence (inverted) and another showing the un-inverted sequence (normal).

After the two reference genomes were created, run BWA on the original fastq files two times, using normal.fasta and inverted.fasta as reference genomes, resulting in two SAM files: normal.sam & inverted.fasta.

g. **Quantify The Inversion**

After mapping to the two reference genomes, the matlab function quantify can be used to extract the ratio inverted/normal genotypes in the clone. Run the command line:

```
Inverted_fraction = quantify_inversion(normal_sam,
inverted_sam)
```

with the filenames of the two SAM files as inputs, to produce the desired ratio.

Provided Matlab Functions:

Function	Inputs	Outputs
`read_sam_file`	`filename`: name of the SAM file.	`data`: a matrix containing the relevant information for further analysis
`detect_inversions`	`data`: a matrix generated by read_sam_file	`location`: a vector containing locations of putative inversions<br>`score`: a vector containing scores for each inversion in location. The highest the score, the more reads align to the putative inversions
`reads_over_region`	`data`: a matrix generated by read_sam_file<br>`center`: the center of the area of interest (use locations generated by detect_inversions to visualize the inversion)<br>`range`: the span of the area of interest. Default = 2000 Kbp	`region`: a small matrix containing information on reads
`find_trimmed_clusters`	`region`: a small matrix containing information on reads, generated by `reads_over_region`.	`location`: a vector of genomic locations<br>`trimmed`: the number of trimmed reads whose trimmed edge maps directly to each genomic location

`prepare_reference_genomes`	`filename`: name of the reference genome fasta file<br>`center`: genomic location signifying the center of the desired reference genomes<br>`region`: number of bps from each side of center to be included in the new reference genomes<br>`left_boundary`: left boundary of the inverted locus<br>`right_boundary`: left boundary of the inverted locus	The function produces two reference genomes as fasta files: `filename_center_normal.fasta` and `filename_center_inverted.fasta`
`quantify_inversion`	`normal_sam`: filename of the SAM output file after mapping the WGS data to the un-inverted (normal) reference genome<br>`inverted_sam`: filename of the SAM output file after mapping the WGS data to the inverted reference genome	`inverted_fraction`: A number signifying the fraction of the inverted genotype of the entire population
